# Verses

**Published:** 1907-01

**Authors:** 


### VERSES.

Success will oft play
But the prelude of sorrow;
The fad of today
Is the jest of tomorrow.—*Exchange.*

Let us drink to the thought that where'er man roves
He is sure to find something blissful and dear,
And that when he is far from the lips that he loves
He can always make love to the lips that are near.

### WHITE vs. BLACK.

W'en dey gits a w'ite man fo' de cote,
Dey sets er jury ob his kine,
An' ef de charge am salls an' batter,
Dey lets 'im off wid er small size fine.

But w'en de cullud gent gits dar,
De same w'ite jury meets his gazė,
An' jes' fer swattun' er nurrer coon
'E j'ines chain-gang for sixty days.

Dere's no use buckin' gin de w'ite
Onless youse daff er color blin',
Fer ef yer do you'll fin' hit true
Youse losin' time erway behin'.

—B. H. Teague.

### A TOAST.

Here's to the stork,
A most valuable bird,
That inhabits the residence districts.
He doesn't sing tunes,
Nor yield any plumes,
But he helps out the vital statistics.

—*Portland Oregonian.*

